# The impact of economic, political and social globalization on overweight and obesity in the 56 low and middle income countries

**DOI:** 10.1016/j.socscimed.2015.03.030

**Published:** 2015-05

**Authors:** Yevgeniy Goryakin, Tim Lobstein, W. Philip T. James, Marc Suhrcke

**Affiliations:** a Health Economics Group, Norwich Medical School, University of East Anglia, Norwich NR4 7TJ, UK; b UKCRC Centre for Diet and Activity Research (CEDAR), Institute of Public Health, Cambridge, UK; c World Obesity Federation, London, UK; d London School of Hygiene and Tropical Medicine, London, UK; e Centre for Health Economics, University of York, York, UK

**Keywords:** Developing countries, Globalization, Obesity, Overweight

## Abstract

Anecdotal and descriptive evidence has led to the claim that globalization plays a major role in inducing overweight and obesity in developing countries, but robust quantitative evidence is scarce. We undertook extensive econometric analyses of several datasets, using a series of new proxies for different dimensions of globalization potentially affecting overweight in up to 887,000 women aged 15–49 living in 56 countries between 1991 and 2009. After controlling for relevant individual and country level factors, globalization as a whole is substantially and significantly associated with an increase in the individual propensity to be overweight among women. Surprisingly, political and social globalization dominate the influence of the economic dimension. Hence, more consideration needs to be given to the forms of governance required to shape a more health-oriented globalization process.

## Highlights

• Globalization as a whole is significantly associated with individual overweight risk among women.
• Social and political globalization have strong positive association with overweight probability among women.
• Women in the most economically globalized countries are less likely to be overweight.

## 1 Introduction

Globalization has often been blamed for the rapid rise in obesity in much of the developing world (Hawkes, 2006; Popkin, 2006; Zimmet, 2000). The existing evidence for this claim does, however, rest primarily on case studies and simple ecological comparisons of national conditions. A notable exception is a recent study by De Vogli et al. (2013) who explored the influence of economic globalization (e.g. foreign direct investment or trade) on obesity world-wide. Arguably, the scarcity of quantitative data amenable to statistical analysis relates to the difficulty in quantifying the complex, multi-faceted nature of globalization. Economists were among the first to try to quantify the different components of globalization in their attempt to assess its impact on economic growth (Dollar and Kraay, 2004; Dreher, 2006). Indeed, the measures of globalization commonly employed have been exclusively economic, commonly proxied by e.g. total imports and exports or foreign direct investment, expressed as a share in GDP. Yet, globalization is not solely an economic process, and even if it were, there is more to economic globalization than the mere flow of goods and capital.

More recent efforts at measuring globalization were built on the conceptualisation by Keohane and Nye (2000) of three different relevant dimensions of globalization: (1) economic: long distance flows of goods, capital and services as well as information and perceptions that accompany market exchanges, (2) political: the diffusion of government policies internationally, and (3) social: the spread of ideas, information, images, and people (Dreher, 2006). Dreher et al. (2008a) have developed the so-called KOF index of globalization to capture each of these dimensions (as well as additional sub-dimensions). For all dimensions, this index was created using comprehensive data collected annually, from 1970 to 2013. In this paper we make use of this new measure and its various components, to arrive at a more detailed and nuanced assessment of the impact of different dimensions of globalization on overweight in low- and middle-income countries.

All three of these components of globalization might have contributed to obesity in low- and middle-income countries, and because they capture different dimensions and – as will be shown further below – are at best imperfectly correlated with each other, it is important to examine the influence of each sub-dimension separately. Taken together, globalization may be contributing to obesity by stimulating increased calorie consumption, and/or smaller energy expenditure. While there exists a considerable literature which considers the role of technological change in affecting energy expenditure and consumption (e.g. (Finkelstein et al., 2005; Huffman and Rizov, 2007; Lakdawalla and Philipson, 2009; Tomas Philipson, 2001; TJ Philipson and Posner, 2003b; Swinburn et al., 2011), the literature that considers the potential globalization & overweight/obesity nexus from the point of view of how globalization affects energy imbalance is quite limited. Nevertheless, as globalization may be both a product and a driver of technological change, they may have similar causal links with overweight through a set of factors collectively known as the “nutritional transition” (Popkin, 2001; Popkin et al., 2012). Specifically, both globalization and technological change may be associated with urbanisation (with living in the cities offering a greater choice of food at lower prices), increasing use of cars and of mechanical aids (resulting in a decline in physical activity), and with a general increase in fat and sugar intake both of which, probably through their effects on energy density, contribute to weight gain (Amine et al., 2002; Hooper et al., 2012; Te Morenga et al., 2013). Thus both technological change and globalization may lead to a lower cost of calorie intake, as well as to the higher opportunity cost of expending calories, resulting in the higher probability of obesity/overweight (TJ Philipson and Posner, 2003a). In the case of globalization, the nutritional transition may also be facilitated by the importation of cheaper, higher energy density foods from the industrialized world, rather than from the countries' internal production.

The most readily recognized manifestation of economic globalization is the opening of markets to foreign trade and investment in the second half of the last century, which entailed a substantial increase in agribusiness-related foreign direct investment (FDI) (Hawkes, 2006). Much of this investment went into food processing (Popkin et al., 2012; Thow, 2009), thus potentially accelerating the nutritional transition and leading to a greater obesity burden (Popkin, 2001, 2006; Popkin et al., 2012).

Political factors relating to the formation of regional trade blocks, or participation in various international treaties, may also have played a role, by acting as a precursor to greater economic integration via the opening of food markets to free trade and consequent nutritional change associated with overweight. On the one hand, greater political integration on a regional level is likely to lead to deeper regional cooperation (e.g. in the form of trade blocks), while on the other hand it may also create mechanisms, for instance, trade barriers, designed to protect participating countries from outside economic competition (Dreher, 2006). While the precise impact of such manifestations of political integration on overweight in developing countries is hard to predict, it may at least be conceivable that political globalization acts independently of (or as a facilitator of) purely economic forces. Differential effects of political vs. economic globalization have, for instance, been found in recent research examining the impact of globalization on economic growth (Dreher, 2006).

Social and cultural globalization, involving cross-border movement of cultures and openness of media, may also have increased a population's perception of the supposed benefits of foreign lifestyles (e.g. in the form of greater car use, decreasing calorie expenditures) as well as of foreign diets (e.g. which may lead to greater calorie consumption through intake of fast food rich in fats and sugars). The effect of social globalization on overweight may therefore be akin to the effect of urbanization on various technologies potentially associated either with the reduction in energy expenditure over time (Monda et al., 2007; Popkin, 1999; Rivera et al., 2002; Swinburn et al., 2011), or with more abundant supply and consumption of cheaper, higher calorie foods (Drewnowski and Popkin, 1997, 1999; Popkin and Gordon-Larsen, 2004).

In addition to examining the importance of these different components of globalization, a further unique feature of our analysis consists of the integration of the various indicators of globalization into a world-wide dataset containing individual-level information up to 887,000 individuals. This allows us to a) utilise information on the (objectively measured) overweight status of each individual and b) to control for relevant individual-level covariates (e.g. education, age, residence, household size) – a feature that should increase analytical precision, compared to the analysis of country-level data alone (which was used by De Vogli et al. (2013)). To better isolate the effect of the various manifestations of globalization, it is important to control for a range of country-level factors that may simultaneously affect individual overweight risk and the country-level indicators of globalization, including the total GDP as a proxy of the size of the market, the Human Development Index, as well as the Index of Economic Freedom from the Heritage Foundation, which measures the quality of economic and legal institutions. Through this analysis we aim to find out whether overall globalization indeed increases the individual likelihood of overweight, and whether the different dimensions of globalization – economic, political and social – play a greater or lesser part in raising the risk of overweight.

## 2 Methods and their rationale

### 2.1 Definition and measurement of the component variables of globalization

Globalization is our independent variable of primary interest. We seek to capture both the influence of globalization as a whole as well as its relevant sub-components: economic, social and political globalization dimensions.

1) Total globalization is measured using the KOF total globalization indicator (Dreher, 2006), which is an aggregation of three sub-components, described below.
2) Economic globalization: Our primary measure of economic globalization is the relevant KOF sub-index, which is a composite measure comprising the following variables: trade (in percent of GDP); foreign direct investment (FDI) stocks (in percent of GDP), portfolio investment (in percent of GDP), income payments to foreign nationals (in percent of GDP), hidden import barriers, mean tariff rate, taxes on international trade (in percent of current revenue) and capital account restrictions.
3) Political globalization: We take advantage of the political KOF index mentioned above, which is a composite measure including information on the following four components: number of foreign embassies in a given country; membership in International Organizations; participation in U.N. Security Council missions; number of signed international treaties (Dreher et al., 2008a). This component is designed to measure the degree of a country's international political engagement (Dreher, 2006). It was used, for instance, in studies examining the influence of globalization on partisan politics (Potrafke, 2009) and government expenditure patterns (Dreher et al., 2008b).
4) Social globalization: Our main measure of this type of globalization is the social KOF globalization index, which is based on the following variables: telephone traffic transfers (percent of GDP); international tourism foreign population (in percent of total population); international letters (per capita); internet users (per 1000 people); TVs (per 1000 people); trade in newspapers (percent of GDP); number of McDonald's restaurants (per capita); number of Ikea (per capita); trade in books (percent of GDP).

### 2.2 Econometric specifications

Starting with the most parsimonious model, we are primarily interested in how individual risk of overweight is affected by various manifestations of globalization:

(1) $$Y_{cit}=X_{ct}\beta +C_{cit}\gamma +D_{t}+e_{cit}$$

where *Y*_*cit*_ is a dummy for being above normal weight (i.e. either overweight or obese), for individual *i* living in country *c* at time *t*; *X*_*ct*_ is a vector of country-level covariates measuring various dimensions of globalization with the corresponding parameter vector *β*; *C*_*cit*_ captures individual-level control variables with the corresponding parameter vector *γ*; *D*_*t*_ is a time effect allowing us to control for potential time dependence or for any world-wide factors (e.g., global economic crises) that could affect our associations of interest, and *e*_*cit*_ is an error term assumed to be uncorrelated with *X*_*(i)ct*_. To account for potential spatial correlation of the error term, all our standard errors are clustered according to cluster IDs provided in the dataset. In the rest of the paper we shall refer to “overweight” when we mean ‘being above normal weight’, i.e. either overweight or obese.

Our data came from several sources. Outcome and individual control variables were obtained from the Demographic and Health Surveys (DHS) collected in a total of 56 countries over the period 1991–2009 (variable definitions, as well as the full list of countries and survey years used is provided in the online Annex Supplementary material). The DHS surveys have been extensively described elsewhere (S. Subramanian et al., 2011). Country level control variables came from World Development Indicators collated by the World Bank. Globalization indices were taken from the KOF globalization index of globalization prepared at the Swiss Federal Institute of Technology (Dreher, 2006). Finally, Economic Freedom Index from the Heritage Foundation was used as an additional control variable.

The outcome variable of interest (i.e. being overweight) was defined as having a body mass index (BMI) greater or equal to 25 kg/m^2^. The BMI was calculated by dividing each person's weight in kilograms by height squared in meters. In order to trim outliers, observations for women whose BMI was above 50 kg/m^2^, or whose weight was either greater than or equal to 220 kg, were excluded. In addition, observations for women whose height was recorded as either greater than or equal to 2.2 m, were also excluded. Overall, in the pooled sample, data on BMI were available for about 72% of the full sample of 1,225,816 women. We restricted the sample for the analysis to non-pregnant women only, aged 15–49 years. Although the original sample contained women who were older than 49, for the vast majority of observations the anthropometric data was collected only in the 15–49 group. The actual sample size used in the regression analysis varied between 756,000 and 887,000.

As an alternative to using overweight as a dependent variable, some studies have employed the continuous variable BMI (De Vogli et al., 2013; S. Subramanian et al., 2011). We decided not to follow this approach, since increases in BMI associated with various independent variables may have very different implications, depending on the initial BMI value. For example, a change in BMI from 18 to 19 (i.e. from being malnourished to having normal weight – i.e. a desirable outcome) can hardly be compared to an increase in BMI from 24 to 25 (i.e. from having normal weight to being overweight – i.e. an undesirable outcome). Measuring the association between covariates and BMI does not capture this difference, while measuring the effect of covariates on overweight (treated as a dummy variable) does.

Globalization-related indicators contained in vector *X*_*(i)ct*_ were defined for one overall proxy of globalization as well as three sub-dimensions: economic, political and social (Dreher, 2006), as discussed above. For each index, and for each year, we split the values for each country into four quartiles to enable a more intuitive interpretation of the resulting parameters on the relationship between overweight and globalization, rather than using the un-transformed or log-transformed KOF-scores. For example, a positive parameter on the second dummy (assuming the first dummy serves as a reference) would suggest an increase in the risk of overweight for people living in a country that is located in the second globalization quartile, relative to other 55 countries in any given year. Using quartiles also allows us to capture potential non-linearities in the relationship between globalization and overweight.

As countries compete for more investment by becoming more open relative to others in a given year (Asiedu, 2002), we have chosen a year-specific categorization for globalization categories. Alternatively, we could have categorized countries based on their position relative to all other countries in all years combined, but that would answer a different question: how becoming more globalized not only relative to each other – but also relative to some long-term average globalization level – is related to overweight risk.

In addition, vector *C*_*cit*_ contains individual-level covariates expected to improve precision in estimating the main vector of parameters, *β*. The vector includes indicators for various levels of education, for different age groups, for living in a city, for occupational status, as well as for family size. Education was defined using DHS dummies for six levels, i.e. for people with no, incomplete primary, complete primary, incomplete secondary, complete secondary and higher education. The occupational status for a woman depended on self-reporting her current employment status, and separate dummies were defined for being unemployed, working in the services sector (professional and managerial; clerical; sales; household and domestic; services), in agriculture (agriculture employed and self-employed) and in a manual (skilled manual; unskilled manual) occupation.

The main requirement for consistent parameter estimation in model (1) is that the error term is uncorrelated with the covariates. This is unlikely to be a reasonable assumption, as both overweight and globalization may be driven by some other unobserved factors not included in model (1). In principle, our dataset allows us to include country fixed effects (CFE), which should control for any time-invariant unobservable drivers of globalization and overweight. However, with country fixed effects included, only across time variation (i.e. “within-variation”) in country-level indicators will be used. For 19 out 56 countries (including the largest country-India), only one year of data was collected, so with the addition of country fixed effects, these countries would drop out of the analysis. Moreover, even in countries that had more than one year of data (n = 37), only a few had any variation in the globalization quartiles across years. In the specifications that include both individual and country controls, only 9–10 countries (out of 56) per globalization dimension had any variation in the globalization quartiles, resulting in a big drop-out of countries from the analysis (including some very large ones, e.g. Brazil, Turkey, Egypt, India, Nigeria, Bangladesh, Ethiopia and the Philippines). As this reduction in the sample is attributable to our transformation of the globalization indices into dummy variables (which we adopted for ease of interpretability of the resulting coefficients), this can be remedied by avoiding the transformation of the globalization indicators and using them as un-transformed variables. Hence, when it comes to the (important) comparison between the OLS- and FE-based results, we will revert to the use of the untransformed variables.

As a first step, we deal with the confounding problem by including a set of country-level covariates contained in vector *C*^*2*^_*ct*_, as in specification (2) below.

(2) $$Y_{cit}=X_{\left(i\right)ct}\beta +C_{cit}^{1}\gamma +C_{ct}^{2}\delta +D_{t}+e_{cit}$$

The choice of the country-level confounders was informed by the existing literature on the factors which facilitate movement of trade and investment between countries, and therefore are drivers of globalization. In addition, these variables are expected to be related to overweight risk. They include the size of the market (Asiedu, 2006; Bevan and Estrin, 2004), measured here as total GDP (taken from WDI). The size of a country's GDP is also likely to be related to the level of economic development, and thus, in turn, may affect the obesity risk (Goryakin and Suhrcke, 2014). In addition, foreign investors may consider it more worthwhile to invest in countries with higher overall levels of education and socioeconomic development (Asiedu, 2006; Walsh and Yu, 2010). The Human Development Index (HDI) developed by UNDP is a well-known metric which takes into account not only living standards as measured by GDP per capita, but also two other important components: life expectancy at birth and the literacy rate. Globerman and Shapiro (2002), for example, found that HDI and FDI were significantly correlated in specifications which did not control for governance institutions and infrastructure indicators. Likewise, it was found in several studies (e.g. Dinsa et al., 2012; Goryakin and Suhrcke, 2014; C. Monteiro et al., 2004b) that socioeconomic status and development may be related to overweight and obesity, and therefore controlling for Human Development Index appears to be important in this case.

In addition, another important determinant of globalization (and potentially of economic and social development, which in turn may affect overweight prevalence independently of globalization) is the quality of economic and legal institutions (Asiedu, 2006; Obwona, 2001; Walsh and Yu, 2010). In this paper, we utilize the Index of Economic Freedom from the Heritage Foundation. It takes into account a number of factors potentially important in the decision-making by foreign investors to engage in economic relationships with countries, such as: a quantitative measure of the ability to start, operate, and close a business; absence of tariff and non-tariff barriers; measure of the tax burden imposed by government; security of property rights; freedom from corruption; flexibility of the labour markets. Therefore this indicator is likely to be particularly valuable in our search for relevant proxies for drivers of country-level globalization.

As we mentioned above, although the above approach is designed to control for a range of potentially important confounders, not taking advantage of the within-country variation, when such option is in principle available, would be too costly. Therefore, as a final check, we also conduct country fixed effects estimations on the untransformed globalization scores. Although parameter interpretation is more difficult in this case, there is much more within-variation when untransformed scores are used, and this allows us to test whether findings from the OLS estimation will also hold when controlling for potential time invariant, unobserved country-level confounding.

The authors of the study did not have to obtain ethical approval, as they only analysed secondary, fully anonymized individual-level data from the publicly available Demographic and Health Surveys, as well as some country-level data.

## 3 Results

### 3.1 Description of main variables

In the annex Table S1, we present overweight prevalence by country and year (Supplementary material). In most countries where there were at least two years worth of observations, overweight prevalence tended to increase over the years, although at different rates. Overweight prevalence was generally considerably higher in Eastern Mediterranean countries, and was the lowest in Africa and South East Asia.

Table S2 shows how the total globalization score varied in each country by year (Supplementary material). In almost all countries, the value of the score increased, although again, the rate of change did differ. In Table S3, the relative ranking of the countries by year is set out, according to the same score (Supplementary material). It is evident that the most globalized countries (e.g. Turkey, Brazil, Egypt, Jordan) tended to remain the most globalized in most years, while the same consistency was true for the least globalized countries (e.g., Central African Republic, Congo Democratic Republic, Chad). There appeared to be more variation in relative ranking for countries that were in between these two extremes, although in most cases the rate of change in the score was modest.

**Fig. 1:**
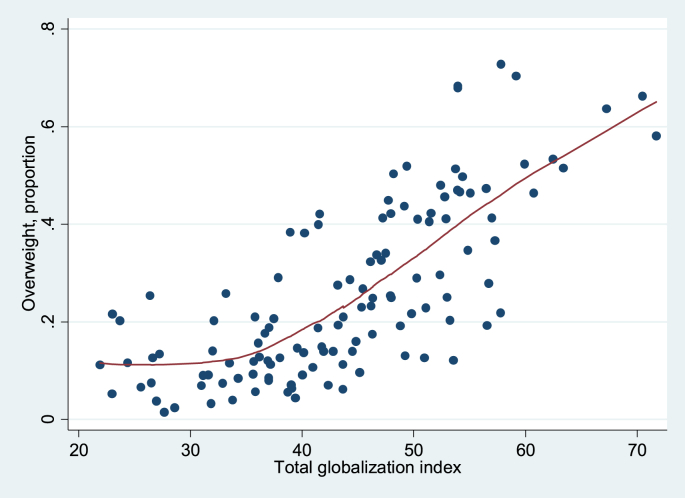
Lowess, unconditional association between overweight and total globalization index, 1991–2009. Source: DHS dataset; KOF index. Bandwidth = 0.8.

**Fig. 2:**
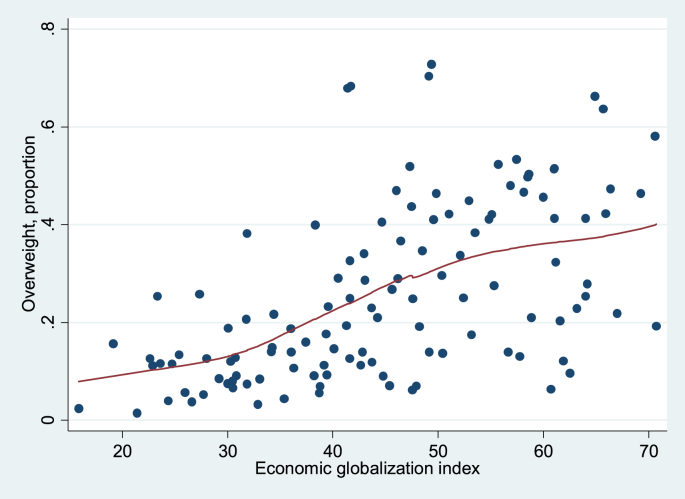
Lowess, unconditional association between overweight and economic globalization index, 1991–2009. Source: DHS dataset; KOF index. Bandwidth = 0.8.

**Fig. 3:**
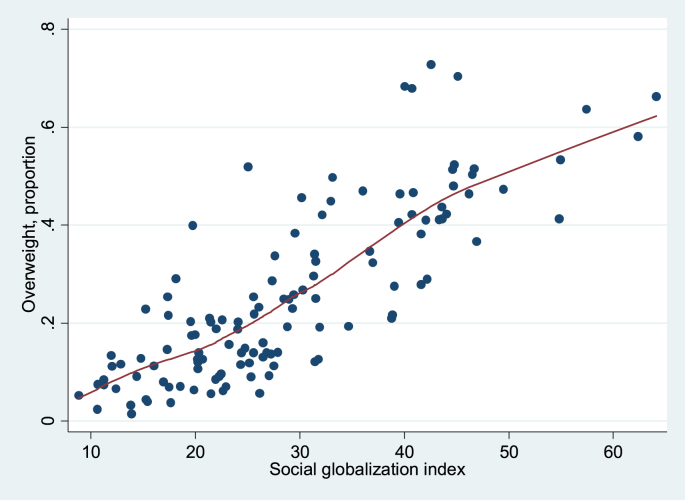
Lowess, unconditional association between overweight and social globalization index, 1991–2009. Source: DHS dataset; KOF index. Bandwidth = 0.8.

**Fig. 4:**
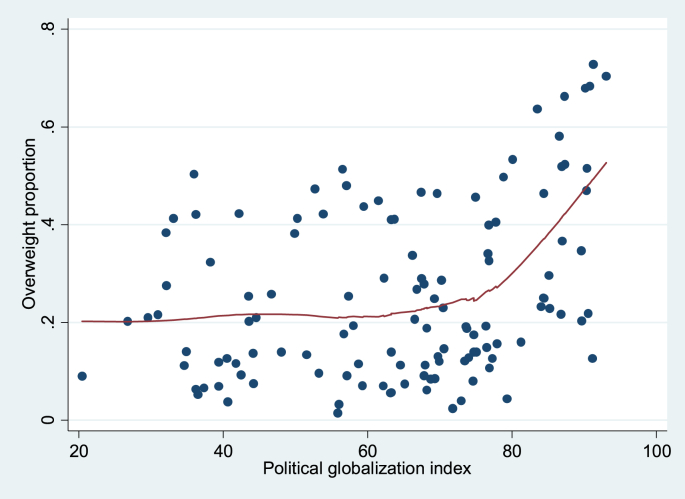
Lowess, unconditional association between overweight and political globalization index, 1991–2009. Source: DHS dataset; KOF index. Bandwidth = 0.8.

Finally, Figs. 1–4 show local regression graphs plotting non-parametric relationships between each globalization score and overweight prevalence in each country. These figures reveal that the relationship appears positive, quite pronounced and mostly linear for the social globalization score. On the other hand, it appears considerably weaker for the economic score. For total and political scores, the relationship seems quite strong, but mostly non-linear. In the former case, it seems that the association is flat for the least globalized countries, before becoming strongly positive. For the political dimension, it appears that there is no relationship to overweight for the majority of countries, except for the most globalized ones, for which we observe a strongly positive association.

### 3.2 Overall globalization

Table 1 sets out the association between overweight and the overall globalization index, split into 4 quartiles. In the first column, not controlling for any covariates except for time dummies and the Sub-Saharan Africa dummy, we find that living in the countries which are in the top quartile for this metric is related to a 29.2 percentage points (p.p.) greater risk of being overweight, compared to the reference category of living in countries with the lowest quartile of the total globalization index. There is also a visible gradient: each higher total globalization quartile is associated with a greater overweight risk, with the shape suggesting a convex pattern. However, as this association may in part be driven by country-level confounding, it is also important to consider its robustness by including relevant controls. In column 2, the adding of individual control variables improves the precision of the estimates, while also somewhat reducing the magnitude of the association. What matters more, however, is the addition of the country level controls: results in column 3 demonstrate that their addition further reduces the magnitude of the association, although the parameters for the globalization dummies remain significant and positive.

Looking at the effect of the main control variables (Table 1, column 3), women with no education are significantly less likely to be overweight than women with the most education; the risk of being overweight increases with age; women that are unemployed or in service occupations and reside in urban areas are more likely to be overweight. Women with no children are less likely to be overweight than women with 6 or more children, whereas women with 1–5 children were more overweight than those with 6 or more children. Moreover, an increase in the size of the market (i.e. total GDP) by 1 billion dollars is associated with an about 0.02 p.p smaller risk of overweight. With HDI ranging from 0 to 1, an increase by 0.1, for example, is associated with about an 8 p.p. greater risk of being overweight. Interestingly, better economic and legal institutions have an opposite effect: an increase of the score by 1 is related to an about 0.5 p.p. smaller overweight risk, suggesting that our proxies for economic and social development on the one hand, and for the quality of economic and legal institutions the other hand, are controlling for two distinct sources of potential confounding.

### 3.3 Sub-components of globalization

Prior to entering into the regression results, we determined whether each of the sub-components of globalization indeed captured distinct phenomena. As shown by the cross-correlation matrix, the sub-components are not too closely correlated with each other (Table 2), except for economic and social components. The correlation is particularly weak between political and economic globalization (r = 0.15), underlining the need to “unpack” the overarching concept of globalization into its constituent parts.

#### 3.3.1 Economic globalization

The first three columns in Table 3 assess the influence of economic globalization on overweight. The results in column 1 without controls for any factors except time dummies and a sub-Saharan African dummy, indicate that greater economic globalization is associated with a greater risk of being overweight. Adjusting for individual covariates, however, reduces the magnitude of the association. The biggest impact on parameter sign, however, occurs after adding country controls: now the relationship becomes concave, with people living in the most economically globalized countries having *lower* probability of being overweight, although this finding needs to be seen in the light of the very small magnitude of this association (i.e. only about 1p.p. lower probability).

#### 3.3.2 Political globalization

Columns 4–6 in Table 3 provide the results on the role of political globalization in affecting individual chances of being overweight. In the basic specification, (column 4), the relationship appears convex, with a fall in the probability of being overweight in the second and third quartile, before an increase for the most politically globalized countries (column 4). However, the addition of individual, and especially country level controls, leads to a more pronounced association: column 6 shows that people living in the most politically globalized countries have a 13.5p.p. greater risk of being overweight, compared to people living in the least globalized countries. This is also true for people living in the third quartile, although the increase in the probability of overweight is considerably smaller.

#### 3.3.3 Social globalization

In columns 7–9 of Table 3 we consider the association between social globalization and overweight. It appears that this dimension has the most stable and pronounced association with overweight across dimensions, as adding different sets of control variables changes the magnitude of the association only slightly. People living in the most socially globalized quartile have an about 18 p.p. greater risk of being overweight, compared to the least globalized group.

### 3.4 All globalization indices combined

Next, we consider the association between overweight and all globalization indices taken together. One potential disadvantage of this approach is some collinearity between different sub-components (especially between social and economic dimensions, as shown in Table 2). On the other hand, putting these scores together in the same model may help ensure an additional degree of control for residual confounding. As the results in column 10 of Table 3 reveal, this approach turns the negative association between economic globalization and overweight into a more pronounced one, while making little difference for the political and social components.

### 3.5 Robustness checks

Some of our findings may be partly driven by the differences in sample size across specifications. For example, the sample size of the basic specifications in Table 3 is up to 887,000, while it is 765,000 in the most adjusted specifications. To check the robustness of the results we estimated the regression parameters for identical samples in Table 4, for each of the three globalization types. Comparing the estimates from Tables 3 and 4 confirms that there is little difference in the size of the economic and social globalization parameters, implying that changes in parameter size across specifications are not due to the differences in sample size. On the other hand, for political globalization, the relationship with overweight becomes uniformly positively signed in all 3 specifications in the identical samples (columns 4–6 in Table 4).

Earlier in the paper, the analysis with OLS using globalization scores transformed into quartiles was presented as this allowed a more intuitive interpretation of results. However, we recognize that this approach is costly, as it effectively precludes a country fixed effects analysis (which would allow controlling for an important source of unobserved confounding) due to a very small within-variation. So to complete our analysis, both OLS and FE estimates (with the same set of control variables as in columns 3, 6, 9 in Table 4) are presented using the original, untransformed globalization scores. Even though interpretation of our key parameter estimates now becomes less clear, this comparison is useful in that it allows us to examine whether the OLS findings continue to hold when the assumption of no correlation between globalization scores and time-invariant unobservables is relaxed.

From Table 5, we can see that that when the globalization dimensions are combined in column 5 – arguably the most comprehensive specification – the signs are identical in both OLS and CFE models, and the magnitudes of the effects of economic and political globalization are at least close to each other. We also see that the magnitude of the CFE associations remains substantive. For example, a 50 percentage point (p.p.) increase in overall globalization score entails an about 15 p.p. greater overweight risk. This compares with an about 16.8 p.p. greater overweight risk for the most globalized countries relative to the least globalized ones (Table 1, column 3). This change in magnitude is not dramatically different, when comparing the results between Tables 5 and 3 for other dimensions.

We also estimate overweight as a quadratic polynomial function of globalization dimensions (results not shown here, but available on request). In order to ensure better interpretability and to mitigate the multicollinearity problem, we centred our estimation on the mean values of the globalization dimension scores. We found the main parameters to be virtually identical for all dimensions. In addition, there appears to be a convex relationship between total and political globalization and overweight, a mostly linear negative relationship between economic globalization and overweight, and a mostly linear positive association between social globalization and overweight.

## 4 Discussion

While most of the existing literature focussed on the relationship between economic globalization and obesity, specific quantitative measures of the range of potentially very different globalization-related drivers involved have not been examined previously. In this analysis we find that the relationship between overweight and globalization depends on the specific dimension of globalization. Thus, while both political and (especially) social globalization dimensions appear strongly positively related to the greater overweight risk, the same is not apparent for economic globalization.

More concretely, comparing different dimensions of globalization and including suitable adjustments for confounders and covariates we find for the first time that political and social globalization consistently show a positive association with the individual odds of overweight: in our preferred specification (i.e. with country controls), the risk of being overweight among women is about 13.5 p.p. (or 17.8 p.p.) greater in the most politically globalized group (or in the most socially globalized group) compared to the least globalized group. This finding is also confirmed in the models using the untransformed globalization scores, although the magnitude of the association is notably smaller for the social (but not for the political) dimension in the CFE compared to the OLS model. Although arguably the biggest attention has so far been directed at the impact of economic globalization, we have found that living in the most economically globalized quartile of countries predicts a 1 p.p. smaller overweight risk than in the least economically globalized ones. This is a rather surprising finding, given the focus of most of the literature on the potential link between obesity and economic globalization (Hawkes, 2006), and the scant attention paid to other dimensions. This finding is also slightly at odds with recent results by De Vogli et al. (2013), who found – using aggregate cross-country level data rather than individual level data – that national BMI (as opposed to overweight) was significantly positively related to the KOF index for economic globalization in 127 countries. Having said that, the parameter sign for the economic dimension was quite sensitive to the inclusion of country-level controls. This appears to be consistent with the hypothesis that at least part of the relationship between economic globalization and overweight may be driven by country-specific factors such as economic development and infrastructure, education, attractiveness of economies to investors, as well as the size of the market.

Inevitably, our study suffers from several limitations. Most of them are data-related, and are thus similar to those faced by other studies which also used DHS data to examine correlates of obesity (Goryakin and Suhrcke, 2014; Monteiro et al., 2004a; Subramanian et al., 2010). For example, the sample was necessarily restricted to women only, and mostly of child-bearing age. Therefore, generalizing our findings to women of all age groups, let alone to both genders, is not possible. Nevertheless, since the age group of 15–49 represents the most productive group of women, who also typically have a number of dependants, focussing attention on this demographic segment may be warranted for informing policies to tackle overweight. Most importantly, we are limited in drawing major causal claims about our findings, especially in relation to 19 countries that were only present in the sample for one year (and thus could not provide any within-variation for the fixed effects analysis).

There are a few other intrinsic data-related concerns which call for caution when interpreting the findings. Thus, by the nature of the research frame, most sampled women were mothers with at least one child under 5 years of age (Monteiro et al., 2004a). This is potentially problematic in that such women may more likely be overweight, although the reverse may be true in the lowest income countries, where both pregnancy and breastfeeding may lead to large energy needs relative to family resources (and thus potentially to malnourishment). Nevertheless, keeping with Monteiro et al. (2004a) assessment, this should not make a substantial difference in terms of the *association* between overweight and the extent of globalization, especially given that we are controlling for the number of children in our analysis as well as for the educational level of mothers.

Another problem is that very few countries stayed in the sample for all periods, given the nature of the DHS data collection. Whereas in some countries (e.g. Egypt, Ghana) data was collected every five years or even more frequently, in many others it was collected for no more than two years. In 19 countries, data was only available for one year. There was also very little variation in our categorical globalization variable across years, which prevented us from undertaking country fixed effects analysis using the globalization indicator dummies. Nevertheless, when using untransformed globalization scores as exposure variables, our country fixed effects findings were mostly in line with our earlier OLS estimates presented in Tables 1, 3 and 4.

It remains possible, however, that some time-varying variables (which country fixed effects cannot control for) may still be a source of bias for our results. For example, availability of infrastructure, wars, economic shocks and famine may affect both the extent of globalization and overweight risk. However, although we are not controlling for these factors explicitly, we nevertheless control for the Human Development Index, as well as the Index of Economic Freedom (which proxies for the quality of economic and legal institutions). Both of these variables, in our view, should to a large extent account for such confounders.

Finally, in this paper, we have only considered the contemporaneous association of globalization with overweight/obesity. It is possible that it may operate with some time lag, but there was little variation across time for globalization indices, and therefore the effect of time lags is unlikely to be estimated with any precision, if the distributed lag model (as seems appropriate) is used.

While these results cannot be given a causal interpretation, they do provide evidence of statistically significant positive association between some dimensions of globalization and overweight. If more robust statistical evidence were found on the causal link between globalization and obesity, what might appropriate policy responses be? It bears emphasising that such evidence would not imply that it would be appropriate to halt or slow down the progress of globalization, but the challenge would be to find ways of limiting and countering the adverse health consequences of globalization while preserving its beneficial effects. Having said that, not all types of globalization appear to affect the risk of obesity equally: the economic dimension, for example, appears to do less harm than previously thought and social and other changes stemming for politically related factors seem of greater importance.

These conclusions have two implications. First, more research is needed to understand the ways in which social and political globalization – as well as economic – influence overweight. The composite elements within the globalization indices could be examined to identify those which are most closely related to overweight risk. For example, it would be useful to know if the increase in McDonald's outlets, an arguably more direct index of the availability of energy dense diets, is more closely associated with the development of overweight than the increase in IKEA outlets, and if the former retains its association after controlling for the latter. Similarly, what are the key political factors – are they related to market freedoms or to democratic expression or to the adoption of current Western political attitudes – and how do these interact with economic and cultural/social factors? Open societies and cultural globalization go hand in hand with open markets and open media, with rapid penetration of advertising and brand promotion by global corporations, together with the depiction of supposedly desirable Western lifestyles which in turn help create a merging of food environments and food cultures as globalization progresses.

Secondly, with greater clarity about the key aspects of globalization becoming available, the challenge to public health policy becomes better focused. For example, if it is shown that fast food outlets are closely associated with overweight prevalence, then what are the policy implications? Is the fast food chain itself a problem, or does it simply reflect the composite effects of FDI policy and cultural openness to advertising and brand promotion, or a more direct effect of a closely related factor, such as a rise in soft drinks consumption (Basu et al., 2013)? Increasing attention is being paid by health promoters to the role of transnational corporations (Hastings, 2012) and accumulating evidence that the rate of increase in consumption of unhealthy food products parallels that of tobacco and alcohol and is fastest in low- and middle-income countries (Stuckler et al., 2012). This has led to public health policy analysts calling for public regulation and market intervention to prevent the harm caused (Moodie et al., 2013), and international agencies have, for example, made recommendations to limit children's exposure to the advertising of unhealthy foods (PAHO, 2011; World Health Organization, 2010). While these policy proposals are widely discussed in the public health arena, they remain marginal to the larger discussions on economic growth and global development. Thus there was no expression of the need to tackle the negative health effects of globalization in the Millennium Development Goals (UN, 2000) which are due to expire in 2015. The High Level Panel steering the post-2015 Sustainable Development programme has yet to specify their target areas for action, but of the 27 international members, 10 have economics, trade and finance backgrounds, three have private sector experience (including one with experience of working for Unilever and Nestlé) but none appears to have public health experience or health qualification (UN, 2013). If globalization in at least some of its dimensions is having a significant impact on the risk of excess weight, then there is indeed a need for stronger governance mechanisms able to take responsibility for protecting health during the globalizing process as recently highlighted by the Oslo/Lancet Commission (Ottersen et al., 2014).

## Figures and Tables

**Table 1:** The relationships between the index of total globalization and overweight in women aged 15–49, Ordinary least squares (OLS) regression results.

	Baseline	Individual controls	Individual and country controls
	(1)	(2)	(3)
Total globalization quartile 2	0.076*** (0.003)	0.054*** (0.002)	0.048*** (0.003)
Total globalization quartile 3	0.125*** (0.003)	0.063*** (0.002)	0.014*** (0.004)
Total globalization quartile 4	0.292*** (0.003)	0.225*** (0.003)	0.168*** (0.004)
No education	–	−0.097*** (0.003)	−0.087*** (0.003)
Incomplete primary education	–	−0.024*** (0.003)	−0.032*** (0.003)
Complete primary education	–	−0.008*** (0.003)	−0.015*** (0.003)
Incomplete secondary education	–	−0.016*** (0.002)	−0.023*** (0.003)
Complete secondary	–	0.045*** (0.003)	0.029*** (0.003)
15–24 years	–	−0.231*** (0.002)	−0.236*** (0.002)
25–34 years	–	−0.124*** (0.001)	−0.125*** (0.001)
0 children	–	−0.063*** (0.002)	−0.055*** (0.002)
1–2 children	–	0.020*** (0.002)	0.023*** (0.002)
3–5 children	–	0.039*** (0.002)	0.042*** (0.002)
Service occupation	–	0.024*** (0.001)	0.015*** (0.002)
Agriculture occupation	–	−0.080*** (0.002)	−0.080*** (0.002)
Manual occupation	–	−0.021*** (0.002)	−0.019*** (0.002)
Urban	–	0.092*** (0.002)	0.086*** (0.002)
GDP, billions, constant$			−0.0002*** (0.000)
HDI			0.809*** (0.015)
Economic Freedom Score			−0.005*** (0.000)

N observations	887,409	864,949	756,345
R-squared	0.111	0.203	0.229

Cluster-robust standard errors in parentheses. Sample restricted to women aged 15–49. No controls (except time dummies and Saharan African dummy) are included in the baseline specification. Reference categories for each of the sets of dummy variables: living in the least globalized quartile of countries, women with higher education, aged 35–49, having 6 or more children, being unemployed, and living in a rural location. All specifications contain time dummies. ***p < 0.01, **p < 0.05, *p < 0.1.

**Table 2:** Correlation matrix of each dimension of globalization, 1991–2009.

	Total	Economic	Social	Political
Total	1.00	–	–	–
Economic	0.81	1.00	–	–
Social	0.84	0.71	1.00	–
Political	0.62	0.15	0.23	1.00

**Table 3:** The relationship between economic, political and social globalization and overweight in women aged 15–49 years, OLS regression results.

	Baseline	Individual controls	All controls	Baseline	Individual controls	All controls	Baseline	Individual controls	All controls	All controls
	(1)	(2)	(3)	(4)	(5)	(6)	(7)	(8)	(9)	(10)
Econ globalization, quartile 2	0.117*** (0.003)	0.079*** (0.002)	0.093*** (0.002)							0.034*** (0.003)
Econ globalization, quartile 3	0.147*** (0.003)	0.086*** (0.003)	−0.007** (0.003)							−0.039*** (0.004)
Econ globalization, quartile 4	0.139*** (0.003)	0.080*** (0.003)	−0.010*** (0.003)							−0.039*** (0.003)
Political globalization, quartile 2				−0.016*** (0.003)	0.001 (0.003)	0.002 (0.003)				0.003 (0.003)
Political globalization, quartile 3				−0.026*** (0.004)	−0.005* (0.003)	0.012*** (0.003)				0.025*** (0.003)
Political globalization, quartile 4				0.043*** (0.004)	0.038*** (0.003)	0.135*** (0.004)				0.117*** (0.004)
Social globalization, quartile 2							0.048*** (0.003)	0.027*** (0.002)	0.028*** (0.002)	0.022*** (0.002)
Social globalization, quartile 3							0.191*** (0.003)	0.150*** (0.002)	0.196*** (0.003)	0.181*** (0.004)
Social globalization, quartile 4							0.279*** (0.003)	0.205*** (0.003)	0.178*** (0.004)	0.195*** (0.004)

N observations	876,355	856,291	756,345	887,409	864,949	756,345	887,409	864,949	756,345	756,345
R-squared	0.089	0.186	0.222	0.079	0.181	0.222	0.109	0.197	0.224	0.234

Cluster-robust standard errors in parentheses. Sample restricted to women aged 15–49. No controls (except time dummies and sub Saharan African dummy) are included in the baseline specification (columns 1, 4, and 7). In columns 2, 5 and 8, controls also include education, age, number of children, occupation and urban residence dummies. In columns 3, 6, 9, 10, the following controls are also added: total GDP (constant 2000 dollars); Human Development Index, Economic Freedom score. ***p < 0.01, **p < 0.05, *p < 0.1.

**Table 4:** Robustness checks: identical sample size across specifications.

	Baseline	Individual controls	All controls	Baseline	Individual controls	All controls	Baseline	Individual controls	All controls
	(1)	(2)	(3)	(4)	(5)	(6)	(7)	(8)	(9)
Econ globalization, quartile 2	0.131*** (0.003)	0.088*** (0.002)	0.093*** (0.002)						
Econ globalization, quartile 3	0.165*** (0.004)	0.101*** (0.003)	−0.007** (0.003)						
Econ globalization, quartile 4	0.119*** (0.003)	0.067*** (0.003)	−0.010*** (0.003)						
Political globalization, quartile 2				0.011** (0.004)	0.014*** (0.003)	0.002 (0.003)			
Political globalization, quartile 3				0.013** (0.005)	0.013*** (0.004)	0.012*** (0.003)			
Political globalization, quartile 4				0.063*** (0.005)	0.046*** (0.004)	0.135*** (0.004)			
Social globalization, quartile 2							0.031*** (0.003)	0.017*** (0.003)	0.028*** (0.002)
Social globalization, quartile 3							0.226*** (0.003)	0.172*** (0.003)	0.196*** (0.003)
Social globalization, quartile 4							0.305*** (0.003)	0.231*** (0.003)	0.178*** (0.004)

N observations	756,345	756,345	756,345	756,345	756,345	756,345	756,345	756,345	756,345
R-squared	0.091	0.192	0.222	0.081	0.188	0.222	0.117	0.208	0.224

Cluster-robust standard errors in parentheses. Sample restricted to women aged 15–49. No controls (except time dummies and sub Saharan African dummy) are included in the baseline specification (columns 1, 4, and 7). In columns 2, 5 and 8, controls also include education, age, number of children, occupation and urban residence dummies. In columns 3, 6 and 9, the following controls are also added: total GDP (constant 2000 dollars); Human Development Index, Economic Freedom score. ***p < 0.01, **p < 0.05, *p < 0.1.

**Table 5:** Robustness checks: estimating the relationship between overweight and globalization using original globalization scores.

	(1)	(2)	(3)	(4)	(5)
	**OLS-based estimates**				
Total globalization	0.010*** (0.000)				
Economic globalization		0.001*** (0.000)			−0.001*** (0.000)
Social globalization			0.009*** (0.000)		0.010*** (0.000)
Political globalization				0.003*** (0.000)	0.003*** (0.000)
Observations	756,345	756,345	756,345	756,345	756,345
R-squared	0.224	0.215	0.228	0.219	0.233
	**Estimates using country fixed effects**				
Total globalization	0.003*** (0.001)				
Economic globalization		−0.0016*** (0.000)			−0.001* (0.000)
Social globalization			0.001* (0.001)		0.001** (0.001)
Political globalization				0.002*** (0.000)	0.002*** (0.000)
Observations	756,345	756,345	756,345	756,345	756,345
R-squared	0.100	0.100	0.100	0.100	0.100

Cluster-robust standard errors in parentheses. Sample restricted to women aged 15–49. The following controls are added in all specifications: age, number of children, occupation and urban residence dummies, Saharan African dummy, total GDP (constant 2000 dollars); Human Development Index, Economic Freedom score. ***p < 0.01, **p < 0.05, *p < 0.1.
