# Supplementary material for: The impact of economic, political and social globalization on overweight and obesity in the 56 low and middle income countries
Source: Soc Sci Med. 2015 May;133:67–76. doi: 10.1016/j.socscimed.2015.03.030 (PMC4416723; doi:10.1016/j.socscimed.2015.03.030)
Supplement: Supplementary file 1 [file mmc1.docx]

Annex S1. Overweight prevalence by country and year. 1991-2009

|  | 1991 | 1992 | 1993 | 1994 | 1995 | 1996 | 1997 | 1998 | 1999 | 2000 | 2001 | 2002 | 2003 | 2004 | 2005 | 2006 | 2007 | 2008 | 2009 |
| --- | --- | --- | --- | --- | --- | --- | --- | --- | --- | --- | --- | --- | --- | --- | --- | --- | --- | --- | --- |
| Armenia |  |  |  |  |  |  |  |  |  | 0.41 |  |  |  |  | 0.42 |  |  |  |  |
| Azerbaijan |  |  |  |  |  |  |  |  |  |  |  |  |  |  |  | 0.47 |  |  |  |
| Bangladesh |  |  |  |  |  |  | 0.02 |  |  | 0.04 |  |  |  | 0.08 |  |  | 0.11 |  |  |
| Benin |  |  |  |  |  | 0.09 |  |  |  |  | 0.19 |  |  |  |  | 0.19 |  |  |  |
| Burkina Faso |  |  | 0.07 |  |  |  |  |  | 0.06 |  |  |  | 0.09 |  |  |  |  |  |  |
| Bolivia |  |  |  | 0.34 |  |  |  | 0.47 |  |  |  |  | 0.46 |  |  |  |  | 0.50 |  |
| Brazil |  |  |  |  |  | 0.35 |  |  |  |  |  |  |  |  |  |  |  |  |  |
| Cambodia |  |  |  |  |  |  |  |  |  | 0.06 |  |  |  |  | 0.10 |  |  |  |  |
| Cameroon |  |  |  |  |  |  |  | 0.21 |  |  |  |  |  | 0.29 |  |  |  |  |  |
| CAR |  |  |  | 0.07 |  |  |  |  |  |  |  |  |  |  |  |  |  |  |  |
| CDR |  |  |  |  |  |  |  |  |  |  |  |  |  |  |  |  | 0.11 |  |  |
| Chad |  |  |  |  |  |  | 0.05 |  |  |  |  |  |  | 0.07 |  |  |  |  |  |
| Colombia |  |  |  |  | 0.41 |  |  |  |  | 0.41 |  |  |  |  | 0.41 |  |  |  |  |
| Comoros |  |  |  |  |  | 0.20 |  |  |  |  |  |  |  |  |  |  |  |  |  |
| Congo Brazzaville |  |  |  |  |  |  |  |  |  |  |  |  |  |  | 0.25 |  |  |  |  |
| Cote d’Ivoire |  |  |  | 0.14 |  |  |  |  | 0.19 |  |  |  |  |  |  |  |  |  |  |
| Dominican Rep. | 0.26 |  |  |  |  | 0.38 |  |  |  |  |  |  |  |  |  |  |  |  |  |
| Egypt |  | 0.52 |  |  | 0.47 |  |  |  |  | 0.68 |  |  | 0.68 |  | 0.73 |  |  | 0.70 |  |
| Ethiopia |  |  |  |  |  |  |  |  |  | 0.03 |  |  |  |  | 0.04 |  |  |  |  |
| Gabon |  |  |  |  |  |  |  |  |  | 0.29 |  |  |  |  |  |  |  |  |  |
| Ghana |  |  | 0.13 |  |  |  |  | 0.16 |  |  |  |  | 0.25 |  |  |  |  | 0.30 |  |
| Guatemala |  |  |  |  | 0.34 |  |  |  | 0.44 |  |  |  |  |  |  |  |  |  |  |
| Guinea |  |  |  |  |  |  |  |  | 0.12 |  |  |  |  |  | 0.14 |  |  |  |  |
| Haiti |  |  |  | 0.12 |  |  |  |  |  | 0.25 |  |  |  |  |  | 0.21 |  |  |  |
| Honduras |  |  |  |  |  |  |  |  |  |  |  |  |  |  |  | 0.46 |  |  |  |
| India |  |  |  |  |  |  |  |  |  |  |  |  |  |  |  | 0.13 |  |  |  |
| Jordan |  |  |  |  |  |  | 0.53 |  |  |  |  | 0.64 |  |  |  |  | 0.58 |  | 0.66 |
| Kazakhstan |  |  |  |  | 0.38 |  |  |  | 0.32 |  |  |  |  |  |  |  |  |  |  |
| Kenya |  |  | 0.14 |  |  |  |  | 0.15 |  |  |  |  | 0.23 |  |  |  |  | 0.25 |  |
| Kyrgyzstan |  |  |  |  |  |  | 0.28 |  |  |  |  |  |  |  |  |  |  |  |  |
| Lesotho |  |  |  |  |  |  |  |  |  |  |  |  |  | 0.42 |  |  |  |  |  |
| Liberia |  |  |  |  |  |  |  |  |  |  |  |  |  |  |  |  | 0.20 |  |  |
| Madagascar |  |  |  |  |  |  | 0.04 |  |  |  |  |  |  | 0.07 |  |  |  | 0.06 |  |
| Malawi |  | 0.09 |  |  |  |  |  |  |  | 0.12 |  |  |  | 0.14 |  |  |  |  |  |
| Mali |  |  |  |  |  | 0.08 |  |  |  |  | 0.15 |  |  |  |  | 0.17 |  |  |  |
| Moldova |  |  |  |  |  |  |  |  |  |  |  |  |  |  | 0.41 |  |  |  |  |
| Morocco |  | 0.33 |  |  |  |  |  |  |  |  |  |  | 0.37 |  |  |  |  |  |  |
| Mozambique |  |  |  |  |  |  | 0.09 |  |  |  |  |  | 0.14 |  |  |  |  |  |  |
| Namibia |  | 0.21 |  |  |  |  |  |  |  |  |  |  |  |  |  |  | 0.28 |  |  |
| Nepal |  |  |  |  | 0.01 |  |  |  |  | 0.06 |  |  |  |  |  | 0.09 |  |  |  |
| Nicaragua |  |  |  |  |  |  |  | 0.42 |  |  | 0.48 |  |  |  |  |  |  |  |  |
| Niger |  | 0.08 |  |  |  |  |  | 0.07 |  |  |  |  |  |  |  | 0.13 |  |  |  |
| Nigeria |  |  |  |  |  |  |  |  | 0.23 |  |  |  | 0.20 |  |  |  |  | 0.22 |  |
| Peru | 0.40 |  |  |  |  | 0.45 |  |  |  |  |  |  |  |  |  |  |  |  |  |
| Philippines |  |  | 0.19 |  |  |  |  |  |  |  |  |  |  |  |  |  |  |  |  |
| Rwanda |  |  |  |  |  |  |  |  |  | 0.13 |  |  |  |  | 0.11 |  |  |  |  |
| Senegal |  |  | 0.16 |  |  |  |  |  |  |  |  |  |  |  | 0.22 |  |  |  |  |
| Sierra Leone |  |  |  |  |  |  |  |  |  |  |  |  |  |  |  |  |  | 0.29 |  |
| South Africa |  |  |  |  |  |  |  | 0.51 |  |  |  |  |  |  |  |  |  |  |  |
| Swaziland |  |  |  |  |  |  |  |  |  |  |  |  |  |  |  | 0.50 |  |  |  |
| Tanzania | 0.11 |  |  |  |  | 0.13 |  |  |  |  |  |  |  | 0.18 |  |  |  |  |  |
| Togo |  |  |  |  |  |  |  | 0.11 |  |  |  |  |  |  |  |  |  |  |  |
| Turkey |  |  | 0.46 |  |  |  |  | 0.52 |  |  |  |  |  | 0.51 |  |  |  |  |  |
| Uzbekistan |  |  |  |  |  | 0.22 |  |  |  |  |  |  |  |  |  |  |  |  |  |
| Zambia |  | 0.14 |  |  |  | 0.13 |  |  |  |  |  | 0.12 |  |  |  |  | 0.19 |  |  |
| Zimbabwe |  |  |  | 0.23 |  |  |  |  | 0.27 |  |  |  |  |  | 0.25 |  |  |  |  |

Annex S2. Total globalization score by country and year. 1991-2009

|  | 1991 | 1992 | 1993 | 1994 | 1995 | 1996 | 1997 | 1998 | 1999 | 2000 | 2001 | 2002 | 2003 | 2004 | 2005 | 2006 | 2007 | 2008 | 2009 |
| --- | --- | --- | --- | --- | --- | --- | --- | --- | --- | --- | --- | --- | --- | --- | --- | --- | --- | --- | --- |
| Armenia | 30.7 | 32.3 | 33.2 | 35.3 | 36.5 | 38.0 | 39.3 | 41.8 | 44.3 | 47.2 | 47.4 | 46.2 | 47.6 | 49.5 | 51.5 | 52.7 | 52.8 | 53.0 | 54.3 |
| Azerbaijan | 27.9 | 32.1 | 32.7 | 31.4 | 35.0 | 38.4 | 40.0 | 40.9 | 41.3 | 45.1 | 46.2 | 48.6 | 50.4 | 53.0 | 56.3 | 56.5 | 58.5 | 57.6 | 56.9 |
| Bangladesh | 22.4 | 23.6 | 23.0 | 25.4 | 26.0 | 27.7 | 28.6 | 29.4 | 31.6 | 33.8 | 34.6 | 35.4 | 35.4 | 37.0 | 38.1 | 39.5 | 41.0 | 40.9 | 40.7 |
| Benin | 24.8 | 24.0 | 24.6 | 25.9 | 31.3 | 31.6 | 32.7 | 34.3 | 35.5 | 36.7 | 37.0 | 36.7 | 37.4 | 40.5 | 40.6 | 41.4 | 45.7 | 44.4 | 44.3 |
| Burkina Faso | 30.1 | 27.9 | 31.0 | 32.5 | 32.6 | 32.4 | 34.0 | 39.4 | 38.7 | 38.6 | 38.6 | 39.1 | 40.0 | 40.4 | 41.3 | 42.2 | 43.1 | 43.4 | 44.9 |
| Bolivia | 40.7 | 40.3 | 41.0 | 46.7 | 43.0 | 44.1 | 48.4 | 54.1 | 55.5 | 55.5 | 54.8 | 52.3 | 52.8 | 54.4 | 54.9 | 55.4 | 54.7 | 54.4 | 53.8 |
| Brazil | 48.9 | 50.7 | 52.2 | 52.4 | 53.5 | 54.9 | 55.4 | 55.4 | 56.4 | 55.9 | 58.4 | 58.6 | 57.7 | 58.7 | 59.0 | 58.8 | 59.7 | 58.7 | 59.4 |
| Cambodia | 23.3 | 24.3 | 24.7 | 27.9 | 29.4 | 32.0 | 32.1 | 34.4 | 36.8 | 39.0 | 39.0 | 40.3 | 39.2 | 39.8 | 45.1 | 47.4 | 47.6 | 47.7 | 46.8 |
| Cameroon | 29.9 | 36.6 | 35.2 | 30.3 | 30.8 | 33.2 | 33.9 | 37.5 | 39.7 | 39.5 | 39.2 | 41.4 | 41.4 | 44.3 | 43.7 | 42.7 | 44.4 | 45.5 | 45.8 |
| CAR | 22.0 | 22.2 | 23.0 | 25.6 | 25.6 | 25.5 | 26.5 | 26.4 | 26.0 | 27.1 | 27.2 | 26.8 | 26.9 | 26.8 | 31.6 | 35.0 | 32.3 | 33.4 | 35.9 |
| CDR | 24.1 | 23.8 | 23.1 | 24.5 | 26.4 | 28.8 | 28.0 | 29.2 | 29.6 | 30.0 | 30.9 | 31.7 | 33.0 | 33.2 | 30.8 | 30.6 | 37.2 | 38.3 | 36.6 |
| Chad | 19.9 | 20.1 | 21.4 | 27.4 | 27.4 | 22.6 | 23.0 | 28.6 | 30.4 | 25.6 | 27.4 | 33.1 | 33.2 | 39.0 | 38.8 | 39.8 | 40.0 | 39.2 | 41.0 |
| Colombia | 42.2 | 47.5 | 48.0 | 47.5 | 51.4 | 48.4 | 53.7 | 49.8 | 50.2 | 50.4 | 51.3 | 51.3 | 53.1 | 53.4 | 52.9 | 56.7 | 57.5 | 56.0 | 56.3 |
| Comoros | 22.5 | 22.9 | 23.1 | 23.7 | 24.1 | 23.7 | 24.0 | 24.1 | 25.5 | 25.9 | 27.2 | 27.2 | 28.4 | 27.5 | 28.6 | 29.6 | 30.3 | 30.9 | 31.6 |
| Congo Brazzaville | 37.2 | 36.6 | 38.6 | 40.6 | 41.1 | 41.1 | 41.8 | 40.9 | 43.5 | 39.6 | 39.4 | 40.5 | 41.2 | 46.7 | 47.9 | 46.8 | 44.8 | 44.6 | 51.5 |
| Cote d’Ivoire | 32.0 | 31.6 | 30.9 | 32.0 | 32.8 | 33.5 | 34.1 | 41.3 | 43.2 | 43.7 | 43.8 | 45.0 | 44.7 | 44.9 | 45.2 | 46.1 | 47.9 | 47.8 | 47.9 |
| Dominican Rep. | 33.2 | 33.7 | 35.3 | 35.0 | 35.3 | 40.2 | 41.3 | 42.9 | 45.9 | 52.2 | 54.5 | 51.3 | 51.7 | 56.1 | 56.3 | 59.2 | 60.5 | 55.4 | 55.1 |
| Egypt | 47.8 | 49.4 | 48.9 | 54.0 | 53.9 | 52.8 | 52.5 | 52.9 | 53.3 | 54.0 | 54.5 | 54.6 | 53.9 | 55.2 | 57.8 | 58.5 | 59.5 | 59.2 | 59.4 |
| Ethiopia | 26.2 | 26.2 | 26.2 | 31.3 | 32.0 | 27.7 | 28.9 | 30.0 | 30.6 | 31.8 | 32.4 | 32.2 | 37.2 | 39.3 | 39.4 | 39.4 | 39.3 | 38.3 | 37.2 |
| Gabon | 46.5 | 46.6 | 46.8 | 47.8 | 46.9 | 44.9 | 43.8 | 50.8 | 51.3 | 50.3 | 46.3 | 45.3 | 44.2 | 50.7 | 51.1 | 52.6 | 53.3 | 53.9 | 55.5 |
| Ghana | 34.6 | 35.5 | 38.0 | 39.4 | 39.5 | 43.1 | 42.9 | 44.8 | 45.8 | 49.7 | 49.8 | 50.9 | 53.0 | 55.4 | 54.3 | 52.4 | 54.1 | 52.4 | 54.9 |
| Guatemala | 40.4 | 45.2 | 41.6 | 45.6 | 47.5 | 49.2 | 46.0 | 47.6 | 49.2 | 49.3 | 50.5 | 50.9 | 50.9 | 57.3 | 59.3 | 60.4 | 61.2 | 61.2 | 60.9 |
| Guinea | 35.3 | 33.5 | 32.6 | 32.0 | 32.9 | 33.0 | 34.0 | 36.3 | 36.9 | 39.1 | 40.6 | 39.3 | 41.9 | 37.8 | 41.9 | 42.8 | 43.2 | 44.0 | 45.7 |
| Haiti | 24.4 | 24.2 | 25.4 | 24.4 | 25.4 | 24.9 | 24.7 | 24.0 | 25.3 | 26.4 | 25.3 | 29.0 | 32.2 | 32.7 | 35.6 | 35.8 | 36.1 | 36.3 | 36.6 |
| Honduras | 38.4 | 43.2 | 43.9 | 45.4 | 50.5 | 51.8 | 52.3 | 52.8 | 53.4 | 53.6 | 54.1 | 56.3 | 57.8 | 58.2 | 59.2 | 60.8 | 61.6 | 62.2 | 61.4 |
| India | 33.4 | 35.0 | 35.9 | 36.5 | 38.2 | 41.9 | 42.5 | 42.9 | 43.6 | 44.8 | 45.5 | 46.2 | 47.2 | 47.4 | 49.4 | 50.9 | 52.0 | 52.0 | 51.9 |
| Jordan | 54.7 | 55.1 | 56.4 | 56.0 | 56.7 | 61.0 | 62.5 | 62.2 | 64.1 | 65.8 | 67.1 | 67.3 | 68.1 | 68.2 | 69.6 | 70.0 | 71.7 | 71.5 | 70.5 |
| Kazakhstan | 30.0 | 31.6 | 32.5 | 35.4 | 38.9 | 40.1 | 41.8 | 42.6 | 46.1 | 49.9 | 51.3 | 51.9 | 52.7 | 53.2 | 54.8 | 54.5 | 58.2 | 58.1 | 59.1 |
| Kenya | 37.3 | 39.3 | 42.8 | 43.0 | 43.0 | 42.0 | 42.5 | 41.8 | 43.3 | 44.0 | 44.5 | 45.5 | 46.2 | 47.1 | 47.1 | 47.4 | 48.4 | 48.0 | 49.4 |
| Kyrgyzstan | 30.1 | 33.4 | 34.4 | 37.0 | 39.9 | 40.1 | 43.2 | 47.7 | 50.8 | 53.6 | 52.2 | 51.6 | 49.4 | 51.9 | 52.4 | 56.6 | 58.7 | 56.6 | 56.1 |
| Lesotho | 35.5 | 35.7 | 35.2 | 36.6 | 38.3 | 39.1 | 39.6 | 40.0 | 40.4 | 40.9 | 40.2 | 41.8 | 41.6 | 41.6 | 41.4 | 41.1 | 41.2 | 46.5 | 42.0 |
| Liberia | 27.0 | 28.2 | 28.5 | 29.0 | 30.0 | 30.1 | 29.9 | 29.8 | 30.4 | 30.5 | 30.7 | 29.3 | 29.7 | 29.3 | 30.9 | 31.6 | 32.1 | 32.4 | 33.2 |
| Madagascar | 23.9 | 23.7 | 23.9 | 25.0 | 25.8 | 26.1 | 26.9 | 27.6 | 30.0 | 31.8 | 33.3 | 33.1 | 34.0 | 42.3 | 40.5 | 42.4 | 42.9 | 43.7 | 43.9 |
| Malawi | 30.3 | 31.1 | 29.1 | 37.4 | 36.5 | 33.5 | 27.9 | 30.4 | 35.5 | 35.7 | 38.1 | 38.1 | 39.1 | 40.1 | 39.7 | 39.9 | 41.0 | 43.8 | 40.7 |
| Mali | 26.1 | 25.7 | 29.7 | 33.5 | 33.6 | 34.3 | 34.3 | 35.1 | 36.8 | 37.2 | 39.6 | 42.6 | 43.5 | 44.4 | 43.5 | 46.3 | 44.8 | 44.9 | 46.5 |
| Moldova | 30.4 | 32.4 | 32.3 | 34.8 | 37.4 | 42.0 | 46.6 | 50.1 | 50.3 | 52.5 | 52.1 | 51.8 | 57.3 | 57.1 | 57.0 | 59.3 | 62.0 | 61.5 | 60.9 |
| Morocco | 42.7 | 47.1 | 48.6 | 48.5 | 48.0 | 44.6 | 45.7 | 45.3 | 46.7 | 51.7 | 54.5 | 55.8 | 57.3 | 55.8 | 58.9 | 58.5 | 60.3 | 60.3 | 61.0 |
| Mozambique | 30.3 | 32.5 | 33.1 | 33.7 | 35.2 | 33.0 | 35.6 | 36.0 | 42.0 | 44.1 | 44.6 | 45.9 | 44.5 | 47.3 | 48.2 | 48.7 | 49.3 | 48.8 | 49.0 |
| Namibia | 43.3 | 43.7 | 48.5 | 42.9 | 44.4 | 50.4 | 51.1 | 52.4 | 52.2 | 52.7 | 53.7 | 55.2 | 53.7 | 56.7 | 55.4 | 54.1 | 56.7 | 56.4 | 55.7 |
| Nepal | 24.1 | 25.2 | 26.2 | 26.8 | 27.6 | 29.9 | 31.2 | 31.6 | 32.0 | 35.8 | 36.2 | 36.6 | 34.8 | 35.1 | 36.8 | 37.0 | 36.9 | 36.9 | 37.4 |
| Nicaragua | 38.2 | 37.9 | 39.2 | 38.7 | 41.5 | 42.7 | 44.5 | 47.9 | 51.2 | 52.9 | 52.4 | 53.2 | 54.4 | 53.8 | 52.3 | 54.5 | 54.9 | 54.3 | - |
| Niger | 25.5 | 26.5 | 26.4 | 32.9 | 32.8 | 27.6 | 31.9 | 32.9 | 31.6 | 32.5 | 31.8 | 32.5 | 32.5 | 33.3 | 33.7 | 36.2 | 35.0 | 35.5 | 38.2 |
| Nigeria | 45.1 | 46.7 | 48.8 | 48.3 | 48.7 | 49.5 | 50.9 | 50.4 | 51.1 | 52.5 | 52.1 | 51.4 | 53.3 | 52.7 | 54.5 | 55.7 | 58.4 | 57.8 | 58.0 |
| Peru | 41.5 | 42.5 | 40.0 | 41.3 | 42.9 | 47.7 | 49.4 | 50.1 | 51.2 | 56.6 | 56.8 | 56.4 | 57.4 | 58.8 | 60.3 | 61.7 | 64.3 | 64.8 | 64.5 |
| Philippines | 43.2 | 47.7 | 48.8 | 49.3 | 50.2 | 46.9 | 48.9 | 49.7 | 54.3 | 56.1 | 57.0 | 57.4 | 56.6 | 59.6 | 59.1 | 58.9 | 58.1 | 56.5 | 56.7 |
| Rwanda | 20.8 | 20.8 | 21.1 | 23.7 | 22.2 | 23.5 | 23.7 | 24.4 | 25.4 | 26.6 | 28.9 | 29.3 | 29.8 | 30.6 | 33.5 | 34.6 | 36.1 | 38.3 | 39.5 |
| Senegal | 35.3 | 35.7 | 36.1 | 38.8 | 40.7 | 41.9 | 42.6 | 44.8 | 46.4 | 47.8 | 46.5 | 49.5 | 50.3 | 51.2 | 49.8 | 51.5 | 52.6 | 53.1 | 54.5 |
| Sierra Leone | 23.4 | 23.3 | 21.8 | 25.9 | 24.3 | 24.2 | 23.5 | 27.8 | 28.5 | 30.1 | 29.4 | 29.9 | 30.3 | 38.0 | 37.9 | 37.5 | 38.3 | 37.9 | 38.6 |
| South Africa | 38.7 | 38.7 | 39.4 | 40.5 | 46.9 | 49.3 | 52.1 | 53.7 | 60.1 | 61.6 | 63.2 | 63.3 | 62.9 | 62.3 | 63.3 | 64.2 | 65.3 | 65.4 | 64.4 |
| Swaziland | 44.3 | 44.1 | 44.1 | 45.2 | 43.3 | 43.9 | 42.3 | 46.6 | 45.7 | 46.9 | 44.5 | 49.0 | 47.7 | 47.7 | 45.6 | 48.2 | 47.2 | 47.4 | 51.7 |
| Tanzania | 21.9 | 23.5 | 23.7 | 23.3 | 27.8 | 27.2 | 28.2 | 29.2 | 30.2 | 31.4 | 31.8 | 32.9 | 34.7 | 36.7 | 37.5 | 37.9 | 39.6 | 38.8 | 39.4 |
| Togo | 35.8 | 34.8 | 38.0 | 41.9 | 41.1 | 42.2 | 41.6 | 43.7 | 46.1 | 43.3 | 40.0 | 39.4 | 43.4 | 44.9 | 47.4 | 48.6 | 48.8 | 47.5 | 47.9 |
| Turkey | 51.1 | 52.8 | 55.1 | 59.6 | 60.3 | 60.6 | 61.8 | 59.9 | 60.5 | 61.4 | 62.8 | 61.7 | 62.6 | 63.4 | 69.5 | 67.8 | 68.7 | 68.9 | 70.0 |
| Uzbekistan | 14.4 | 17.2 | 17.8 | 19.4 | 21.4 | 23.0 | 23.8 | 24.8 | 25.7 | 27.9 | 28.9 | 30.2 | 31.8 | 31.8 | 32.9 | 34.0 | 36.0 | 35.8 | 36.7 |
| Zambia | 45.3 | 42.8 | 46.3 | 46.0 | 46.6 | 49.2 | 49.3 | 49.7 | 50.4 | 52.5 | 52.5 | 53.6 | 53.2 | 56.2 | 54.4 | 55.9 | 56.6 | 55.0 | 53.8 |
| Zimbabwe | 39.6 | 41.8 | 43.8 | 45.3 | 46.8 | 45.5 | 45.5 | 45.8 | 45.5 | 46.7 | 46.1 | 45.4 | 47.0 | 46.9 | 46.3 | 48.0 | 47.7 | 48.9 | 50.9 |

Annex S3. Total globalization quintile by country and year, 1991-2009

|  | 1991 | 1992 | 1993 | 1994 | 1995 | 1996 | 1997 | 1998 | 1999 | 2000 | 2001 | 2002 | 2003 | 2004 | 2005 | 2006 | 2007 | 2008 | 2009 |
| --- | --- | --- | --- | --- | --- | --- | --- | --- | --- | --- | --- | --- | --- | --- | --- | --- | --- | --- | --- |
| Armenia | 2 | 2 | 2 | 2 | 2 | 2 | 2 | 2 | 3 | 3 | 3 | 3 | 3 | 3 | 3 | 3 | 3 | 3 | 3 |
| Azerbaijan | 2 | 2 | 2 | 2 | 2 | 2 | 2 | 2 | 2 | 3 | 3 | 3 | 3 | 3 | 4 | 3 | 4 | 4 | 4 |
| Bangladesh | 1 | 1 | 1 | 1 | 1 | 1 | 1 | 1 | 1 | 1 | 1 | 1 | 1 | 1 | 1 | 1 | 1 | 1 | 1 |
| Benin | 1 | 1 | 1 | 1 | 2 | 2 | 2 | 2 | 2 | 2 | 2 | 2 | 2 | 2 | 2 | 2 | 2 | 2 | 2 |
| Burkina Faso | 2 | 2 | 2 | 2 | 2 | 2 | 2 | 2 | 2 | 2 | 2 | 2 | 2 | 2 | 2 | 2 | 2 | 2 | 2 |
| Bolivia | 4 | 3 | 3 | 4 | 3 | 3 | 4 | 4 | 4 | 4 | 4 | 4 | 3 | 3 | 3 | 3 | 3 | 3 | 3 |
| Brazil | 4 | 4 | 4 | 4 | 4 | 4 | 4 | 4 | 4 | 4 | 4 | 4 | 4 | 4 | 4 | 4 | 4 | 4 | 4 |
| Cambodia | 1 | 1 | 1 | 1 | 1 | 2 | 2 | 2 | 2 | 2 | 2 | 2 | 2 | 2 | 2 | 2 | 2 | 2 | 2 |
| Cameroon | 2 | 3 | 3 | 2 | 2 | 2 | 2 | 2 | 2 | 2 | 2 | 2 | 2 | 2 | 2 | 2 | 2 | 2 | 2 |
| CAR | 1 | 1 | 1 | 1 | 1 | 1 | 1 | 1 | 1 | 1 | 1 | 1 | 1 | 1 | 1 | 1 | 1 | 1 | 1 |
| CDR | 1 | 1 | 1 | 1 | 1 | 1 | 1 | 1 | 1 | 1 | 1 | 1 | 1 | 1 | 1 | 1 | 1 | 1 | 1 |
| Chad | 1 | 1 | 1 | 1 | 1 | 1 | 1 | 1 | 1 | 1 | 1 | 1 | 1 | 1 | 1 | 1 | 1 | 1 | 2 |
| Colombia | 4 | 4 | 4 | 4 | 4 | 4 | 4 | 4 | 3 | 3 | 3 | 3 | 3 | 3 | 3 | 4 | 3 | 3 | 3 |
| Comoros | 1 | 1 | 1 | 1 | 1 | 1 | 1 | 1 | 1 | 1 | 1 | 1 | 1 | 1 | 1 | 1 | 1 | 1 | 1 |
| Congo Brazzaville | 3 | 3 | 3 | 3 | 3 | 3 | 3 | 2 | 2 | 2 | 2 | 2 | 2 | 2 | 3 | 2 | 2 | 2 | 3 |
| Cote d’Ivoire | 2 | 2 | 2 | 2 | 2 | 2 | 2 | 2 | 2 | 2 | 2 | 2 | 2 | 2 | 2 | 2 | 2 | 2 | 2 |
| Dominican Rep. | 3 | 3 | 3 | 2 | 2 | 3 | 2 | 3 | 3 | 3 | 4 | 3 | 3 | 4 | 4 | 4 | 4 | 3 | 3 |
| Egypt | 4 | 4 | 4 | 4 | 4 | 4 | 4 | 4 | 4 | 4 | 4 | 4 | 4 | 3 | 4 | 4 | 4 | 4 | 4 |
| Ethiopia | 2 | 2 | 2 | 2 | 2 | 1 | 1 | 1 | 1 | 1 | 1 | 1 | 2 | 2 | 1 | 1 | 1 | 1 | 1 |
| Gabon | 4 | 4 | 4 | 4 | 4 | 3 | 3 | 4 | 4 | 3 | 3 | 2 | 2 | 3 | 3 | 3 | 3 | 3 | 3 |
| Ghana | 3 | 3 | 3 | 3 | 3 | 3 | 3 | 3 | 3 | 3 | 3 | 3 | 3 | 4 | 3 | 3 | 3 | 3 | 3 |
| Guatemala | 3 | 4 | 3 | 4 | 4 | 4 | 3 | 3 | 3 | 3 | 3 | 3 | 3 | 4 | 4 | 4 | 4 | 4 | 4 |
| Guinea | 3 | 2 | 2 | 2 | 2 | 2 | 2 | 2 | 2 | 2 | 2 | 2 | 2 | 1 | 2 | 2 | 2 | 2 | 2 |
| Haiti | 1 | 1 | 1 | 1 | 1 | 1 | 1 | 1 | 1 | 1 | 1 | 1 | 1 | 1 | 1 | 1 | 1 | 1 | 1 |
| Honduras | 3 | 4 | 4 | 4 | 4 | 4 | 4 | 4 | 4 | 4 | 4 | 4 | 4 | 4 | 4 | 4 | 4 | 4 | 4 |
| India | 3 | 3 | 3 | 3 | 3 | 3 | 3 | 3 | 3 | 3 | 3 | 3 | 3 | 3 | 3 | 3 | 3 | 3 | 3 |
| Jordan | 4 | 4 | 4 | 4 | 4 | 4 | 4 | 4 | 4 | 4 | 4 | 4 | 4 | 4 | 4 | 4 | 4 | 4 | 4 |
| Kazakhstan | 2 | 2 | 2 | 2 | 3 | 2 | 3 | 3 | 3 | 3 | 3 | 4 | 3 | 3 | 3 | 3 | 3 | 4 | 4 |
| Kenya | 3 | 3 | 3 | 3 | 3 | 3 | 3 | 3 | 2 | 2 | 2 | 3 | 3 | 2 | 2 | 2 | 2 | 2 | 2 |
| Kyrgyzstan | 2 | 2 | 2 | 3 | 3 | 3 | 3 | 3 | 4 | 4 | 3 | 3 | 3 | 3 | 3 | 4 | 4 | 4 | 3 |
| Lesotho | 3 | 3 | 3 | 3 | 3 | 2 | 2 | 2 | 2 | 2 | 2 | 2 | 2 | 2 | 2 | 2 | 2 | 2 | 2 |
| Liberia | 2 | 2 | 2 | 2 | 1 | 2 | 1 | 1 | 1 | 1 | 1 | 1 | 1 | 1 | 1 | 1 | 1 | 1 | 1 |
| Madagascar | 1 | 1 | 1 | 1 | 1 | 1 | 1 | 1 | 1 | 1 | 1 | 1 | 1 | 2 | 2 | 2 | 2 | 2 | 2 |
| Malawi | 2 | 2 | 2 | 3 | 2 | 2 | 1 | 1 | 2 | 2 | 2 | 2 | 2 | 2 | 2 | 2 | 2 | 2 | 1 |
| Mali | 2 | 2 | 2 | 2 | 2 | 2 | 2 | 2 | 2 | 2 | 2 | 2 | 2 | 2 | 2 | 2 | 2 | 2 | 2 |
| Moldova | 2 | 2 | 2 | 2 | 2 | 3 | 4 | 4 | 3 | 4 | 3 | 3 | 4 | 4 | 4 | 4 | 4 | 4 | 4 |
| Morocco | 4 | 4 | 4 | 4 | 4 | 3 | 3 | 3 | 3 | 3 | 4 | 4 | 4 | 4 | 4 | 4 | 4 | 4 | 4 |
| Mozambique | 2 | 2 | 2 | 2 | 2 | 2 | 2 | 2 | 2 | 2 | 3 | 3 | 2 | 3 | 3 | 3 | 3 | 3 | 2 |
| Namibia | 4 | 4 | 4 | 3 | 3 | 4 | 4 | 4 | 4 | 4 | 4 | 4 | 4 | 4 | 4 | 3 | 3 | 3 | 3 |
| Nepal | 1 | 1 | 1 | 1 | 1 | 1 | 2 | 2 | 2 | 2 | 2 | 2 | 1 | 1 | 1 | 1 | 1 | 1 | 1 |
| Nicaragua | 3 | 3 | 3 | 3 | 3 | 3 | 3 | 3 | 4 | 4 | 4 | 4 | 4 | 3 | 3 | 3 | 3 | 3 | - |
| Niger | 2 | 2 | 2 | 2 | 2 | 1 | 2 | 2 | 1 | 1 | 1 | 1 | 1 | 1 | 1 | 1 | 1 | 1 | 1 |
| Nigeria | 4 | 4 | 4 | 4 | 4 | 4 | 4 | 4 | 4 | 3 | 3 | 3 | 4 | 3 | 3 | 3 | 4 | 4 | 4 |
| Peru | 4 | 3 | 3 | 3 | 3 | 4 | 4 | 4 | 4 | 4 | 4 | 4 | 4 | 4 | 4 | 4 | 4 | 4 | 4 |
| Philippines | 4 | 4 | 4 | 4 | 4 | 4 | 4 | 4 | 4 | 4 | 4 | 4 | 4 | 4 | 4 | 4 | 3 | 3 | 3 |
| Rwanda | 1 | 1 | 1 | 1 | 1 | 1 | 1 | 1 | 1 | 1 | 1 | 1 | 1 | 1 | 1 | 1 | 1 | 1 | 1 |
| Senegal | 3 | 3 | 3 | 3 | 3 | 3 | 3 | 3 | 3 | 3 | 3 | 3 | 3 | 3 | 3 | 3 | 3 | 3 | 3 |
| Sierra Leone | 1 | 1 | 1 | 1 | 1 | 1 | 1 | 1 | 1 | 1 | 1 | 1 | 1 | 1 | 1 | 1 | 1 | 1 | 1 |
| South Africa | 3 | 3 | 3 | 3 | 4 | 4 | 4 | 4 | 4 | 4 | 4 | 4 | 4 | 4 | 4 | 4 | 4 | 4 | 4 |
| Swaziland | 4 | 4 | 4 | 3 | 3 | 3 | 3 | 3 | 3 | 3 | 2 | 3 | 3 | 3 | 2 | 2 | 2 | 2 | 3 |
| Tanzania | 1 | 1 | 1 | 1 | 1 | 1 | 1 | 1 | 1 | 1 | 1 | 1 | 1 | 1 | 1 | 1 | 1 | 1 | 1 |
| Togo | 3 | 3 | 3 | 3 | 3 | 3 | 3 | 3 | 3 | 2 | 2 | 2 | 2 | 2 | 2 | 3 | 3 | 2 | 2 |
| Turkey | 4 | 4 | 4 | 4 | 4 | 4 | 4 | 4 | 4 | 4 | 4 | 4 | 4 | 4 | 4 | 4 | 4 | 4 | 4 |
| Uzbekistan | 1 | 1 | 1 | 1 | 1 | 1 | 1 | 1 | 1 | 1 | 1 | 1 | 1 | 1 | 1 | 1 | 1 | 1 | 1 |
| Zambia | 4 | 4 | 4 | 4 | 4 | 4 | 4 | 3 | 3 | 4 | 4 | 4 | 4 | 4 | 3 | 3 | 3 | 3 | 3 |
| Zimbabwe | 3 | 3 | 4 | 4 | 4 | 4 | 3 | 3 | 3 | 3 | 3 | 2 | 3 | 2 | 2 | 2 | 2 | 3 | 2 |

Annex S4. Variable definitions.

| Variable name | Type | Source | Additional information |
| --- | --- | --- | --- |
| Overweight | Individual | DHS | Defined as dummy=1 if BMI>=25 kg/m^2^ |
| Education | Individual | DHS | Education. Split into dummies: no education; incomplete primary; complete primary; incomplete secondary; complete secondary; higher education. |
| Urban | Individual | DHS | Urban residence |
| Age | Individual | DHS | Age. Split into 3 categories: 15-24, 25-34, 35-49 years |
| Number of children | Individual | DHS | Split into four categories: 0, 1-2, 3-5, 6 and more children |
| Occupation | Individual | DHS | Split into service, agriculture, manual; unemployed |
| Total globalization index | Country | KOF Index | Split into 4 categories, by quartile |
| Economic globalization index | Country | KOF Index | Split into 4 categories, by quartile |
| Social globalization index | Country | KOF Index | Split into 4 categories, by quartile |
| Political globalization index | Country | KOF Index | Split into 4 categories, by quartile |
| Total GDP | Country | WDI | Total GDP, constant 2000 dollars |
| Human Development Index | Country | UNDP | N/A |
| Economic Freedom index | Country | Heritage Foundation | N/A |

Annex S5. List of countries and survey years used in the analysis.

| Countries | Years |
| --- | --- |
| Armenia | 2000, 2005 |
| Azerbaijan | 2006 |
| Burkina Faso | 1993, 1999, 2003 |
| Bangladesh | 1997, 2000, 2004, 2007 |
| Benin | 1996, 2001, 2006 |
| Bolivia | 1994, 1998, 2003, 2008 |
| Brazil | 1996 |
| Central African Republic | 1994 |
| Congo Democratic Republic | 2007 |
| Cambodia | 2000, 2005 |
| Cameroon | 1998, 2004 |
| Chad | 1997, 2004 |
| Colombia | 1995, 2000, 2005 |
| Comoros | 1996 |
| Congo Brazzaville | 2005 |
| Cote d’Ivoire | 1994, 1999 |
| Dominican Republic | 1991, 1996 |
| Egypt | 1992, 1995, 2000, 2003, 2005, 2008 |
| Ethiopia | 2000, 2005 |
| Gabon | 2000 |
| Ghana | 1993, 1998, 2003, 2008 |
| Guatemala | 1995, 1999 |
| Guinea | 1999, 2005 |
| Haiti | 1994, 2000, 2006 |
| Honduras | 2006 |
| India | 2006 |
| Jordan | 1997, 2002, 2007, 2009 |
| Kazakhstan | 1995, 1999 |
| Kenya | 1993, 1998, 2003, 2008 |
| Kyrgyzstan | 1997 |
| Lesotho | 2004 |
| Liberia | 2007 |
| Madagascar | 1997, 2004, 2008 |
| Malawi | 1992, 2000, 2004 |
| Mali | 1996, 2001, 2006 |
| Moldova | 2005 |
| Morocco | 1992, 2003 |
| Mozambique | 1997, 2003 |
| Namibia | 1992, 2007 |
| Nepal | 1995, 2000, 2006 |
| Nicaragua | 1998, 2001 |
| Niger | 1992, 1998, 2006 |
| Nigeria | 1999, 2003, 2008 |
| Peru | 1991, 1996 |
| Philippines | 1993 |
| Rwanda | 2000, 2005 |
| Senegal | 1993, 2005 |
| Sierra Leone | 2008 |
| South Africa | 1998 |
| Swaziland | 2006 |
| Tanzania | 1991, 1996, 2004 |
| Togo | 1998 |
| Turkey | 1993, 1998, 2004 |
| Uzbekistan | 1996 |
| Zambia | 1992, 1996, 2002, 2007 |
| Zimbabwe | 1994, 1999, 2005 |
